# "Paradoxical” Cerebral Volume Changes in Anti‐Amyloid Immunotherapy Trials

**DOI:** 10.1002/alz.091961

**Published:** 2025-01-09

**Authors:** Nick C Fox, Arnaud Charil, Pallavi Sachdev, Hongmei Niu, Michael C. Irizarry, Steven Hersch, Larisa Reyderman

**Affiliations:** ^1^ UK Dementia Research Institute, Queen Square Institute of Neurology, University College London, London United Kingdom; ^2^ Eisai Inc., Nutley, NJ USA; ^3^ Deep Human Biology Learning (DHBL), Eisai Inc., Nutley, NJ USA

## Abstract

**Background:**

Lecanemab is a humanized IgG1 monoclonal antibody that binds with high affinity to Aβ soluble protofibrils. In two clinical study evaluations of lecanemab, Clarity AD (NCT03887455) and lecanemab phase 2 study (Study 201, NCT01767311), the drug showed statistically significant reduction in disease progression during 18 months of treatment relative to placebo. Anti‐amyloid immunotherapy can result in higher rates of “pseudo‐atrophy” (ie, whole brain volume loss or ventricular enlargement) relative to disease progression observed in placebo‐treated subjects. Herein, we evaluated volumetric MRI changes associated with lecanemab treatment to assess the neurological impact.

**Method:**

Volumetric MRI changes over the duration of the Clarity AD study was compared between placebo and lecanemab‐treated participants (n = 1311). In addition, the association between pseudo‐atrophy and longitudinal changes in tau and amyloid PET were evaluated regionally in a subset of participants in the Clarity AD PET sub‐study (n = 342). The association between volume changes and fluid biomarkers was also investigated.

**Result:**

Volumetric MRI results were inconsistent, with slowing of hippocampal atrophy but greater whole brain volume loss on lecanemab relative to placebo. Volume loss was not associated with worsening in any neurodegenerative biomarkers (plasma NfL, CSF NfL, and t‐tau) or clinical outcomes (CDR‐SB, ADAS‐Cog14, ADCS MCI‐ADL). With disease progression (placebo group), regional atrophy was associated with regional tau accumulation, but was not associated with regional amyloid accumulation. With lecanemab treatment, regional pseudo‐atrophy was associated with amyloid clearance and was not associated with regional tau levels or neuronal loss.

**Conclusion:**

Greater volume loss may be related to reduced amyloid plaques and associated dystrophic neurites as well as inflammation/gliosis. Volume changes are not accompanied with worsening in any neurodegeneration biomarkers and likely do not represent neuronal loss.

